# Selection of four mutant alleles of fatty acid desaturase genes for a stable high oleic and low linolenic acid soybean seed oil trait

**DOI:** 10.1007/s00122-026-05302-w

**Published:** 2026-06-24

**Authors:** Hyun Jo, Aaron Lorenz, Brian Diers, Andrew Scaboo, Vincent Pantalone, Zenglu Li, Kristin Bilyeu

**Affiliations:** 1 Department of Applied Biosciences, Kyungpook National University, Daegu, Republic of Korea; 2 Department of Agronomy and Plant Genetics, University of Minnesota, St Paul, MN USA; 3 Department of Crop Sciences, University of Illinois, Urbana, IL USA; 4 Division of Plant Science and Technology, University of Missouri, Columbia, MO USA; 5 Department of Plant Sciences, University of Tennessee, Knoxville, TN USA; 6 Institute of Plant Breeding, Genetics, and Genomics, and Department of Crop and Soil Sciences, University of Georgia, Athens, GA USA; 7 Plant Genetics Research Unit, USDA/ARS, Columbia, MO USA

## Abstract

**Key message:**

The combination of four variant fatty acid desaturate alleles achieves the desired high oleic and low linolenic acid fatty acid profile in soybean seed oil across U.S. production environments.

**Abstract:**

Soybean is a global crop that represents over half of current world oilseed production. Soybean oil has been utilized in the food system based on the functional and health properties of the fatty acid profile; there is an urgent need to adjust the fatty acids in soybean oil to meet current market demands. The soybean high oleic acid seed oil trait (> 75%) in conjunction with a low linolenic acid trait (< 3%) has emerged as an avenue to improve the oil. The objective of this research was to identify a soybean genotypic group comprised of variant alleles of fatty acid desaturase genes that could meet the target stable seed oil fatty acid thresholds across the U.S. soybean production environments. New soybean genotypes were created with different gene combinations for seed oil fatty acid profile and maturity groups, seeds were produced in their target environments, and fatty acid profiles of the seeds were determined. The results demonstrated that a four-gene combination in soybean genotypes using either of two variant versions of one of the genes achieved both the target thresholds in environments across the U.S. soybean production regions. There were significant differences in individual fatty acid levels in the seed oil when comparing the two four-gene genotypic groups. The impact of this work is the new knowledge to design soybean varieties for U.S. production environments with seed oil fatty acid profiles targeted to meet current market thresholds; the fatty acid profiles obtained can serve as the basis for improved products.

**Supplementary Information:**

The online version contains supplementary material available at 10.1007/s00122-026-05302-w.

## Introduction

Soybean (*Glycine max* [L.] Merr.) is the world’s premier oilseed crop, which has recently accounted for the majority of global oilseed production (http://soystats.com/). On a dry weight basis, soybean seeds contain approximately 40% protein and 22% oil (Medic et al. 2014). Soybean is processed into two main products: Vegetable oil used in foods and industry and meal utilized primarily for livestock rations. The need for heat and oxidative stability is critical for oil used in cooking and frying of foods (Choe and Min 2007). However, a balance between the functionality of a cooking oil and its nutritional and health concerns has been the subject of continued research and the evolution of cooking oils over the past century (Matlock 2022).

One avenue to meet market demands for next-generation cooking oil with positive functional and health attributes is high oleic oil that changes the fatty acid profile of the oil (Matlock 2022). Soybean seed oil contains five primary fatty acids: Two saturated fatty acids (palmitic and stearic acid), one monounsaturated oleic acid, and two polyunsaturated fatty acids (linoleic and linolenic acid) (Wilson 2004). Commodity soybean oil typically contains ~ 26% oleic acid (Liu and White 1992). In regard to the nutritional and health attributes of oils, the U.S. Food and Drug Administration (FDA) supported a qualified health claim that consuming oleic acid in edible oils may reduce the risk of coronary heart disease (FDA 2018). The FDA concluded that oleic acid in edible oils containing at least 70% oleic acid justified the claim, so threshold levels of oleic acid in high oleic soybean seed oil have been unofficially set at 75% (FDA 2018).

Soybean was domesticated from a wild ancestor (*Glycine soja* [Sieb. & Zucc.]) native to temperate East Asia, and both the wild and domesticated species are adapted to generally narrow and distinct latitudes because of the influence of daylength on the life cycle stages of the plant (Jeong et al. 2019). Modern soybean varieties are developed through genetics and breeding strategies and have been categorized in the U.S. by the maturity group (MG) designation that was further refined into the Relative Maturity system (Mourtzinis and Conley 2017). In the U.S., soybean varieties range from the shortest life cycle in very early MG to the longest life cycle in late MG.

A molecular model was developed using the three major soybean maturity genes to predict MGs of soybean genotypes (Jo et al. 2019; Langewisch et al. 2017). Selection of reference or alternate alleles of the *E*1 (Wm82.a2.v1 Glyma.06g207800), *E*2 (Glyma.10g221500), and *E3* (Glyma.19g224200) genes in different combinations can target MGs 00, 0, I, II, III or IV, and V or later (Langewisch et al. 2017). Use of molecular markers to predict MGs simplifies selection for genotypes that can be evaluated in desired regions (Jo et al. 2019).

Commodity soybean oil typically contains 20–30% oleic acid, but high oleic soybean oil has been a target for decades (Knowlton 2022). At least two types of biotechnology-enabled high oleic soybean varieties have been commercialized in limited MGs. Plenish**®** varieties combined the high oleic acid biotechnology trait with conventional genetics for the low linolenic acid trait (less than 3% linolenic acid in the seed oil) to fully meet functional expectations for the oil (Knowlton 2022). There has also been considerable progress to develop conventional high oleic soybean varieties utilizing natural and induced variant alleles of fatty acid desaturase genes that control the seed oil fatty acid profile (Hudson and Hudson 2021). The biology underlying the biotechnology-derived high oleic acid trait and conventional high oleic acid trait is related but distinct (Brink et al. 2014; Pham et al. 2010). We have developed conventional high oleic acid soybean genotypes utilizing both missense and null mutant alleles of the *FAD*2*-*1*A* (Glyma.10g278000) gene along with missense mutant alleles of the *FAD*2*-*1*B* (Glyma.20g111000) gene (Bilyeu et al. 2015a, b; Pham et al. 2011, 2010). Some studies also investigated the ability of additional mutant genes in the fatty acid biosynthesis pathway along with mutant alleles of *FAD*2*-*1*A* and *FAD*2*-*1*B* to develop the high oleic and low linolenic trait combination, but those studies used only small numbers of environments and did not include analysis of the trait targeted to different MGs (Bilyeu et al. 2018; Hagely et al. 2021; McDonald et al. 2023; Pham et al. 2012; Willette et al. 2021). The objective of this research was to determine the fatty acid profile of the seed oil from different variant allele combinations for soybean genotypes adapted and grown in the U.S. soybean production regions.

## Materials and methods

Development of 53 soybean genotypes targeted to distinct MGs with different combinations of the fatty acid desaturase alleles was performed essentially as described previously with molecular marker selection for maturity genes *E*1, *E*2, and *E*3 and fatty acid desaturase alleles (Jo et al. 2019). The original sources and details of alleles for the four fatty acid desaturase genes utilized in this study are provided (Online Resource 1). Molecular marker assays for the fatty acid desaturase gene variant alleles utilized SimpleProbe assays (Online Resource 2), except for the *FAD*3*A* deletion alleles, which were interrogated as previously described (Bilyeu et al. 2006). Molecular selection for the maturity gene alleles was as previously described for *E*1, *E*2, and *E*3 (Langewisch et al. 2017, 2014). The maturity allele donors were PI 556749 (KG30), PI 548379 (Mandarin [Ottawa]), PI 662940 (‘Deuel’), PI 662941 (‘Davison’), ‘Skylla’ (Wang et al. 2006), ‘Jake’ (Wang et al. 2006), and experimental lines R07-128, ‘Osage’, NC-Tinius, as well as PI 603452 and PI 210179 in the pedigree from the University of Arkansas breeding program (Cardinal et al. 2012; Chen et al. 2007); G00-3213 and G00-3880 were maturity allele donors for the University of Georgia breeding program (Shi et al. 2015).

The field studies were conducted in 2015, 2016, and 2017 at St. Paul, Minnesota (MG 0, 00, and I), the Crop Sciences Research and Education Center (South Farms) in Champaign, Illinois (MG II), the Lee Greenley Jr. Memorial Research Farm near Novelty, Missouri (MG III/IV), the South Farm Research Center in Columbia, Missouri (MG III/IV), the Fisher Delta Research, Extension and Education Center in Portageville, Missouri (MG V), the East Tennessee Research and Education Center in Knoxville, Tennessee, (MG V) and at Fayetteville, Arkansas (MG V); studies were conducted in 2016 and 2017 at the University of Georgia Iron Horse Plant Science Farm, Watkinsville, GA and Southwest Research and Education Center, Plains, GA (MG VI/VII). The experiments were conducted with three replications in a randomized complete block design at each location with typical small plots for limited seeds that were not necessarily consistent for individual locations or years. For the South Farm Research Center, Missouri and Champaign, Illinois locations, two or three different planting dates in each year were used as independent environments. Field studies were conducted in 2016–2017 in Athens and Plains, Georgia (MG VI/VII) with two replications in a randomized complete block design at each location. For all environments, plants were harvested by plot, and five seeds per plot were sampled and analyzed as a composite for fatty acids in the seed oil by gas chromatography with an Agilent 6890 system as previously described except using an AT-Silar capillary column (Beuselinck et al. 2006).

## Data analysis

Analysis of variance was conducted over different environments using PROC GLM of SAS (SAS Institute, 2016). Genotypic group was treated as a fixed effect, whereas environment, replication nested within environment, and genotype × environment interaction were treated as random effects. Least-square means were compared using Fisher’s least significant difference (LSD) test at the 5% significance level using SAS. The oleic acid of each genotypic group was analyzed by one-tailed *t*-test (upper-tailed) with a threshold of 75.0% oleic acid content using PROC TTEST of SAS. In addition, the linolenic acid of each genotypic group was analyzed by one-tailed *t*-test (lower-tailed) with a threshold of 3.0% linoleic acid content. Comparison of two genotypic groups for each fatty acid was analyzed using a two-tailed *t*-test. Boxplots were generated using the ggplot2 package implemented in *R* (version 4.1.1). To evaluate the stability of genotypic groups for oleic and linolenic acid concentrations across environments, stability regression coefficients (*b*_E_) were estimated following the method of (Lee et al. 2009; Scherder et al. 2008). The stability coefficient was obtained by regressing the mean fatty acid concentration of each genotypic group in a given environment against the environmental index. The environmental index was calculated as the mean fatty acid concentration of all genotypes in a specific environment minus the overall mean across all environments. Genotypic groups with regression coefficients closer to zero were considered more stable across environments, whereas larger deviations from zero indicated greater environmental sensitivity. Stability regression coefficients were estimated using PROC REG in SAS.

## Results

### Development of soybean varieties targeted to different maturity groups with combinations of mutant fatty acid desaturase alleles

In order to evaluate modified seed oil composition in soybean genotypes adapted to U.S. production environments, we developed experimental soybean genotypes with the high oleic acid trait by molecular marker selections from over 24 populations. Parents were identified that donated variant fatty acid desaturase alleles: two genes for the high oleic acid trait (*FAD*2*-*1*A* and *FAD*2*-*1*B*) as well as either none, or *FAD*3*A* alone, or both *FAD*3*A* and *FAD*3*C* to reduce linolenic acid (Online Resource 1). The other parent was selected to target the desired alleles of one or more of the three major maturity genes [*E*1, *E*2, and *E*3; (Langewisch et al. 2017)]. Two distinct alleles for *FAD*2*-*1*A* were utilized [S117N missense (− 1) or indel (− 2)], but different mutant alleles of *FAD*3*A* or *FAD*3*C* were considered interchangeably; there was only one source of P137R missense alleles utilized for *FAD*2*-*1*B* (Table 1). The combination of variant *FAD*2*-*1*A* and *FAD*2*-*1*B* alleles with variant *FAD*3 genes was prioritized with the aim to achieve both high oleic acid and low linolenic acid in the seed oil. For each segregating population, genotype selection was performed for the desired homozygous fatty acid desaturase alleles. Different maturity groups (MG) were also targeted with genotype selection of maturity gene allele combinations based on the established model (Langewisch et al. 2017). The result was an incomplete matrix of soybean genotypes with different combinations of genes for high oleic acid and low linolenic acid seed oil with maturity gene combinations targeted to MG 00, 0, I, II, III/IV, V, and VI/VII. Regardless of maturity group genotypes, we classified these lines into six categories (genotypic group) based on their fatty acid genotype combinations for two to four genes while distinguishing between the missense (S117N) or null (indel) alleles of *FAD*2*-*1*A* (Table 1). A variable number of lines were developed for the different targeted MGs for each genotypic group, so for statistical purposes, the genotypic group was considered the genotype.

**Table 1:** Designation of genotypic group and fatty acid desaturase allele information for soybean germplasm lines developed for different MGs

Genotypic group	*FAD*2*-*1*A*	*FAD*2*-*1*B*	*FAD*3*A*	*FAD*3*C*	# *FAD* genes
HO-1	aa (S117N)	P137R			2
HO-2	aa (indel)	P137R			2
HOL-1	aa (S117N)	P137R	aa^1^		3
HOL-2	aa (indel)	P137R	aa		3
HOLL-1	aa (S117N)	P137R	aa	cc^2^	4
HOLL-2	aa (indel)	P137R	aa	cc	4

^1^*FAD*3*A* variant alleles included splice site, deletion, and W266*, without distinction

^2^*FAD*3*C* variant alleles included G128E and H304Y, without distinction

### Seed oil fatty acid profiles of high oleic soybean lines in different maturity groups

For a total of 21 environments, the soybean lines were grown in field environments appropriate to their targeted maturity gene combinations for three years in Minnesota (MG 00, 0, and I), Illinois (MG II), northern and central Missouri (MG III/IV), and southern Missouri, Tennessee, and Arkansas (MG V); over two years, soybean lines were grown in Georgia (MG VI/VII). Seed samples were evaluated for fatty acid components of the oil. The genotype effect was the most important for oleic, linoleic, and linolenic acid content, although there were also significant effects from environment and genotype × environment interaction (Online Resource 3). None of the environments produced outlier value means for oleic acid or linolenic acid (Online Resource 3). Similarly, each of the maturity group targets produced consistent oleic and linolenic acid values according to the genotypic group (Online Resource 4). Stability regression coefficients (*b*_E_) for oleic and linolenic acid across environments were estimated to evaluate the environmental stability of genotypic groups (Online Resource 5). Based on the stability coefficients, HOLL-2 exhibited the greatest stability for oleic acid concentration across environments, followed by HOL-2 and HO-2, all of which carry the indel null allele of *FAD*2*-*1*A*. For linolenic acid concentration, HOLL-2 also showed the greatest stability across environments, followed by HOLL-1 and HOL-1.

### Seed oil fatty acid profiles of high oleic soybean lines across U.S. production zones

The fatty acid data from the seed oil samples for each genotypic group was averaged for palmitic, stearic, oleic, linoleic, and linolenic acid contents (Table 2). For palmitic acid, there were small significant differences among the genotypic categories, with a range of 7.1 to 7.6%. For stearic acid, there was more variability and significant differences, but the range was 3.4–4.6%. All of the samples had a high oleic acid level, although there were significant differences with a range of 79.8 to 83.6%. The polyunsaturated fatty acids linoleic and linolenic acid each had substantial variation and significant differences among genotypic groups (Table 2).

**Table 2:** Seed oil fatty acid phenotypes for different genotypic groups from combined analyses across environments

Genotypic group	n	Mean ± standard deviation				
		Palmitic acid (%)	Stearic acid (%)	Oleic acid (%)	Linoleic acid (%)	Linolenic acid (%)
HO-1	26	7.1 ± 0.3d^1^	4.6 ± 0.5a	79.8 ± 1.6e	3.8 ± 0.9c	4.7 ± 0.5a
HO-2	278	7.3 ± 0.6c	3.4 ± 0.5d	83.4 ± 1.5a	2.1 ± 0.8e	3.7 ± 0.7b
HOL-1	270	7.5 ± 0.5b	3.5 ± 0.5d	81.2 ± 2.2c	4.7 ± 1.7b	3.1 ± 0.5c
HOL-2	326	7.2 ± 0.6c	3.8 ± 0.6b	83.0 ± 1.5b	3.0 ± 1.1d	2.9 ± 0.5d
HOLL-1	241	7.6 ± 0.6a	3.6 ± 0.6c	80.6 ± 2.6d	6.1 ± 2.0a	2.1 ± 0.3e
HOLL-2	303	7.3 ± 0.6c	3.8 ± 0.5b	83.6 ± 1.3a	3.2 ± 0.8d	2.0 ± 0.2f
LSD (0.05)		0.11	0.10	0.30	0.23	0.08

^1^Different lowercase letters within a column represent significant differences at p < 0.05

### *Null alleles of FAD*2*-*1*A produce higher oleic acid in the seed oil compared to missense FAD*2*-*1*A alleles*

**Fig. 1:**
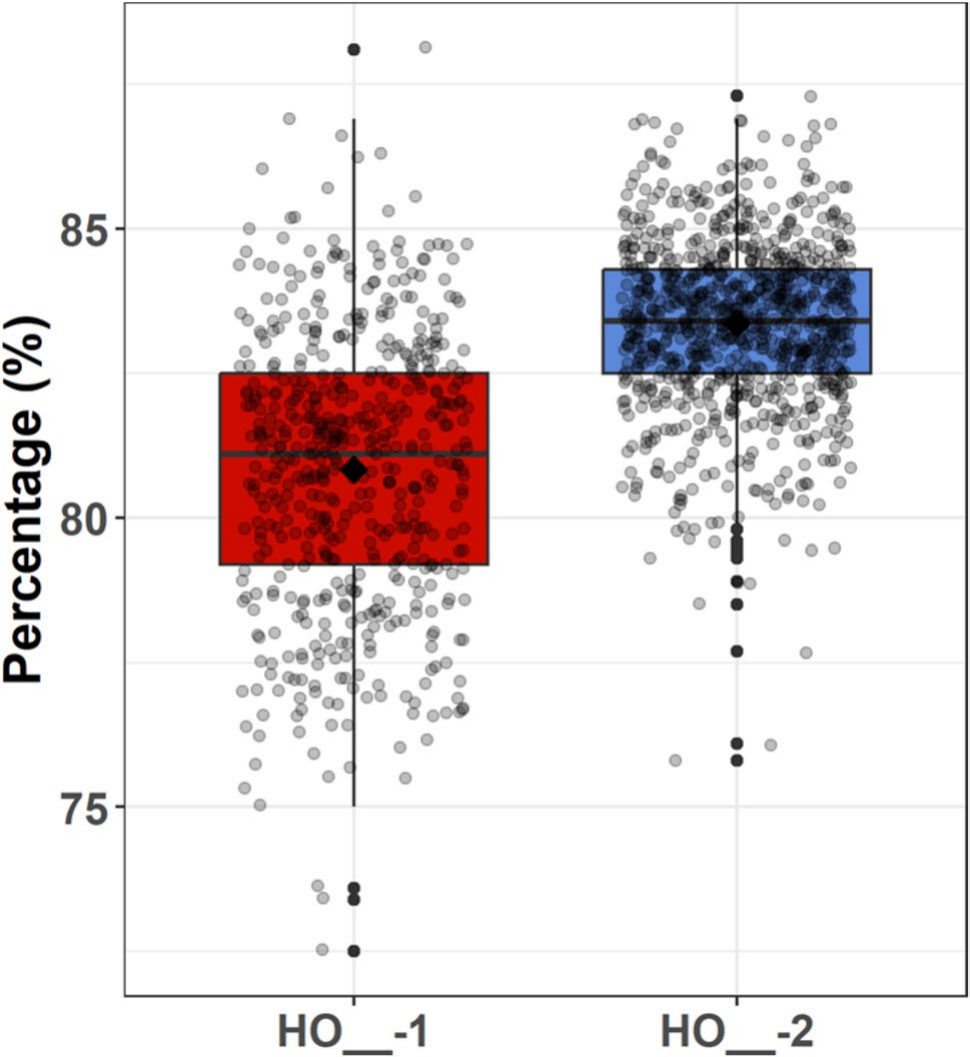
All environment comparison of seed oil oleic acid phenotypes between high oleic samples with the missense or null alleles of *FAD*2*-*1*A*. The percentage of oleic acid in the seed oil is presented in the box and whisker plots for HO__-1 (red box; missense S117N FAD2-1A with missense P137R FAD2-1B; all *FAD3* allele status included) lines and HO__-2 (blue box; null indel FAD2-1A with missense P137R FAD2-1B; all *FAD*3 allele status included) lines ignoring the *FAD*3*A* and *FAD*3*C* allele status. The means are indicated with filled diamonds, the median line transects each box, the quartiles are represented by the upper and lower margins of the box, the whiskers represent range in values, outliers are indicated with black circles, and the individual sample values are represented with gray circles

The samples that contained the missense alleles of *FAD*2*-*1*A* (HO__-1; S117N missense alleles of *FAD*2*-*1*A* regardless of *FAD*3 allele status) were compared with those that contained the null *FAD*2*-*1*A* alleles (HO__-2; indel null alleles of *FAD*2*-*1*A* regardless of *FAD*3 allele status) across all environments (Fig. 1). The mean oleic acid value for HO__-1 was 80.8% versus 83.4% for (HO__-2), a 2.6% difference (*p* < 0.001), where the allelic status of *FAD*3*A* and *FAD*3*C* was ignored (Fig. 1). A difference of ~ 2–4% oleic acid between the missense and null *FAD*2*-A* alleles was present across maturity groups and in each location when both alleles *FAD*2*-*1*A* were tested, except for MG I where the difference was 1% oleic acid (Table 2 and Online Resource 4). In all locations and maturity groups, the HO__-2 samples had higher average oleic acid than the HO__-1 samples.

### Identification of four-gene combinations for a high oleic and low linolenic acid soybean oil trait

**Fig. 2:**
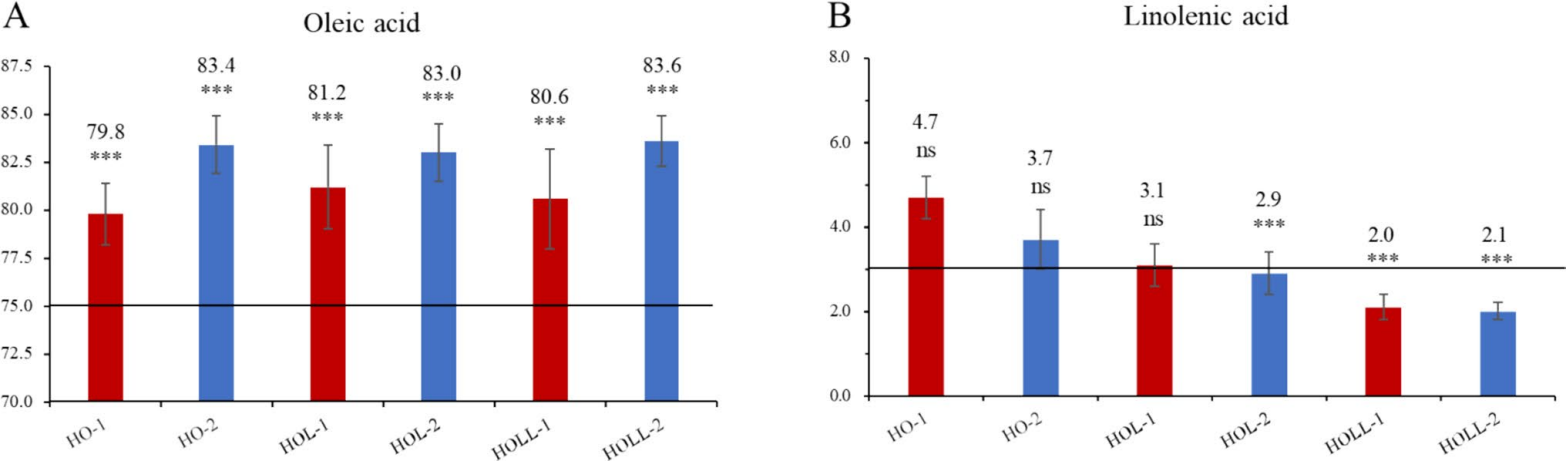
Evaluation of genotypic categories capable of meeting target phenotype thresholds across environments for oleic acid and linolenic acid in the seed oil. Red fill indicates missense S117N FAD2-1A with missense P137R FAD2-1B lines, and blue fill indicates null indel FAD2-1A with missense P137R alleles FAD2-1B lines. **A.** The oleic acid in the seed oil mean percentage histograms and standard deviations (whiskers) were calculated for samples across all environments belonging to the six genotypic categories as described in Table 1. A 75% oleic acid value line represents the threshold for high oleic acid. The means for each genotypic category are indicated above the histogram, and *** indicates significance at the p < 0.001 value for exceeding the threshold. **B.** The linolenic acid in the seed oil mean percentage histograms and standard deviations (whiskers) were calculated for samples across all environments belonging to the six genotypic categories as described in Table 1. A 3% linolenic acid value line represents the threshold for low linolenic acid. The means for each genotypic category are indicated above the histogram, and ns indicates not significantly below the threshold; *** indicates significance at the p < 0.001 value for below the threshold

Developing soybean varieties to meet market expectations for new seed oil composition requires understanding the genetics to achieve the target thresholds for oleic acid (> 75%) and linolenic acid (< 3%) in the seed oil. All of the tested genotypic groups (Table 1) produced significantly higher levels of oleic acid than the threshold across environments (Fig. 2A). Indeed, all but one of the genotypic groups exceeded a mean of 80% oleic acid. However, for the linolenic acid threshold, only soybean genotypes with the HOLL-1 and HOLL-2 four-gene combinations that included both variant *FAD*3 genes produced significantly less than 3% linolenic acid across environments (Fig. 2B). The mean linolenic acid level for the HOL-2 three-gene combination including variant *FAD*3*A* but not *FAD*3*C* was just below 3%; however, the range of linolenic acid values included many samples above 3% for this genotypic group (Fig. 2B).

### *Investigation of seed oil fatty acids for soybean genotypes with four-gene combinations (HOLL-*1* and HOLL-*2*)*

**Fig. 3:**
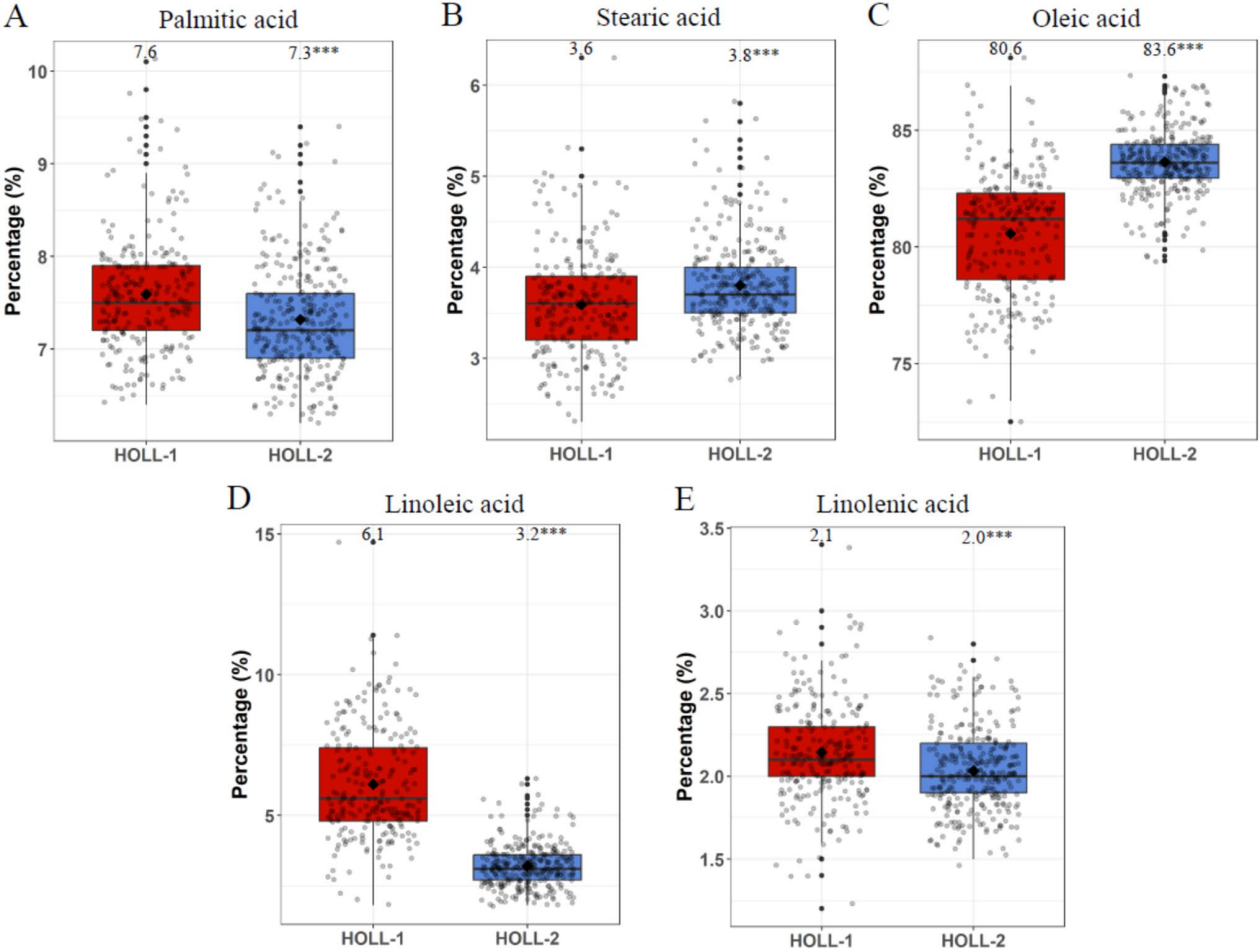
All environment comparison of seed oil fatty acid phenotypes HOLL-1 and HOLL-2 genotypic categories. The percentage of each fatty acid in the seed oil is presented in box and whisker plots for HOLL-1 (red fill) and HOLL-2 (blue fill) genotypic categories that each have a four mutant gene combination (Table 1). The means are indicated with filled diamonds, the median line transects each box, the quartiles are represented by the upper and lower margins of the box, the whiskers represent range in values, outliers are indicated with black circles, and the individual sample values are represented with gray circles. The mean values are indicated above the plots, and *** indicates significant differences at the p < 0.001 value between HOLL-1 and HOLL-2 genotypic category means. A. Palmitic acid. B. Stearic acid. C. Oleic acid. D. Linoleic acid. E. Linolenic acid

The four gene-combinations from genotypic groups HOLL-1 and HOLL-2 (Table 1) were necessary to meet both the oleic acid and linolenic acid oil profile target thresholds across environments. This result informs breeding for high oleic soybean varieties, which can utilize either the missense or indel variant alleles of *FAD*2*-*1*A* along with *FAD*2*-*1*B*, *FAD*3*A* and *FAD*3*C* mutant alleles (Table 1). An evaluation of the five main fatty acids in the seed oil for the HOLL-1 and HOLL-2 soybean genotypes across environments and MGs demonstrated the consistency of the fatty acid profile phenotypes (Fig. 3). The differences between HOLL-1 and HOLL-2 soybean genotypes in mean content of palmitic, stearic, and linolenic acid were each less than 0.5%. The oleic acid difference of 3% is consistent with the analysis above, and the higher linoleic acid level for genotypes with missense alleles of *FAD*2*-*1*A* reflects more desaturation of oleic acid from the weaker missense alleles. Therefore, the fatty acid profile of HOLL-1 seed oil was stable with 11% saturated fats, 81% oleic acid, 6% linoleic acid, and 2% linolenic acid. The fatty acid profile of HOLL version 2 seed oil was stable with 11% saturated fats, 84% oleic acid, 3% linoleic acid, and 2% linolenic acid.

## Discussion

Following their identification and characterization in *Arabidopsis thaliana*, a set of fatty acid desaturase genes became obvious targets to change the seed oil profile in many crop species (Wallis and Browse 2002). Both soybean *FAD*2*-*1 and *FAD*3 gene sets have been characterized independently (He and Traore 2021; Tang et al. 2005). The utilization of dysfunctional variant alleles of fatty acid desaturase genes that control the fatty acid profile in soybean seed oil is now fairly routine, with the *FAD*2*-*1 and *FAD*3 gene natural variants, induced mutant variants, and induced transgenic/genome editing variants, or some combination of these, having achieved commercial production in the U.S. (Knowlton 2022).

The unofficial threshold for oleic acid content in soybean seed oil to achieve the high oleic designation is 75%; there is now a Codex Standard for high oleic soybean oil (HOSO) with a minimum of 65% oleic acid as a percent of total fatty acids in the oil (Codex-Alimentarius 2024). While there is a broad Codex Standard for the linolenic acid content in HOSO (the accepted range is 1% to 6%), prior efforts aimed at commercialization sought to reduce linolenic acid below 3% of the seed oil to provide additional oxidative stability (Knowlton 2022). The commercialized Pioneer® Brand Plenish ® high oleic and low linolenic acid soybean variety portfolio from Corteva has been reported to produce a refined, bleached, and deodorized oil with 75.7% oleic acid and 1.6% linolenic acid (Napolitano et al. 2018). Our results demonstrated slightly higher oleic acid as well as linolenic acid levels. Demand for high oleic soybean varieties is increasing due to benefits to dairy production when high oleic soybeans are part of the ration (Bales and Lock 2024a, b). The United Soybean Board and the US Soybean Export Council funded analyses of the high oleic soybean business and market case to meet growing demand as well as a comprehensive sourcing guide for international customers of high oleic soybeans and soybean oil (https://unitedsoybean.org/wp-content/uploads/2022/12/CLEAN-2230-262-0413-HOSoy-Viability-White-Paper-2023-Supply-Premium-Update-10.14.2022.pdf and https://ussec.org/wp-content/uploads/2025/08/HOSO-Sourcing-Guide-2025.pdf).

Research to develop conventional high oleic and low linolenic acid soybean varieties free from regulatory issues has been primarily done in the public sector with natural and induced variants in the *FAD*2*-*1 and *FAD*3 genes (Hudson and Hudson 2021). While there is no inherent scientific issue with GMO soybeans, the existing regulatory processes in the U.S. and elsewhere provide a more streamlined path to variety release when conventional genetics are utilized or when conventional genetics are combined with a GMO herbicide trait. There have been prior comparisons of the fatty acid profiles of different variant alleles of the *FAD*2*-*1*A* and *FAD*2*-*1*B* genes, either alone, or in combination, and as was expected, a spectrum of oleic acid content can be achieved depending on the alleles utilized (Combs and Bilyeu 2019; Darr et al. 2020; Dierking and Bilyeu 2009; Hoshino et al. 2010; Jo et al. 2022; Lee et al. 2012, 2019; Pham et al. 2011, 2010; Sweeney et al. 2017). Other studies have reported the fatty acid profile in the seed oil for combinations of mutations in soybean *FAD*2*-*1*A* and *FAD*2*-*1*B*, along with *FAD*3*A*, and/or *FAD*3*C* and *FAD*3*B*, though none of those studies spanned multiple maturity groups (Bilyeu et al. 2018; Hagely et al. 2021; Hudson and Carrero-Colón 2025; McDonald et al. 2023; Pham et al. 2012; Willette et al. 2021). Our results show broad maturity group expression of the high oleic and low linolenic acid trait encompassing most of the U.S. soybean production regions.

Here we took a molecular breeding approach to design soybean genotypes with the desired seed composition variant allele combinations targeted to a wide range of maturity groups (Langewisch et al. 2017). This approach successfully achieved the adaptation of the developed soybean genotypes to different U.S. production environments and enabled us to determine the optimized allele combinations to meet unofficial and official thresholds for oleic acid and linolenic acid in the seed oil. Indeed, the soybean high oleic and low linolenic acid trait was demonstrated to be viable with either source of variant *FAD*2*-*1*A* alleles that were investigated in this study; the null allele originating with the PI 603452 (HO__-2) was superior in terms of desired fatty acid profile, with significantly higher oleic acid and lower linolenic acid when combined with *FAD*3*A* and *FAD*3*C* mutant alleles (HOLL-2). This research fulfills a need for information on the viability of the soybean high oleic acid and high oleic with low linolenic acid traits across production environments. Soybean varieties with the ‘Soyleic’ trademark were subsequently developed by public soybean breeders and released in the U.S. with the high oleic acid and low linolenic acid traits conditioned by the alleles characterized in this work.

## Supplementary Information

Below is the link to the electronic supplementary material.

Supplementary file1 (DOCX 28 KB)

## Data Availability

The raw data are available upon request.
