# Supplementary material for: Selection of four mutant alleles of fatty acid desaturase genes for a stable high oleic and low linolenic acid soybean seed oil trait
Source: Theor Appl Genet. 2026 Jun 24;139(7):189. doi: 10.1007/s00122-026-05302-w (PMC13294171; doi:10.1007/s00122-026-05302-w)
Supplement: Supplementary file 1 — Supplementary file1 (DOCX 28 KB) [file 122_2026_5302_MOESM1_ESM.docx]

Online Resource 1

Supplemental Table 1. Soybean fatty acid desaturase gene mutant alleles information

| Wm82.a2.v1 | Gene | Allele | Type | Source | Reference |
| --- | --- | --- | --- | --- | --- |
| Glyma.10g278000 | *FAD2-1A* | S117N (-1) | Missense | 17D | (Pham et al. 2010) |
|  |  | indel (-2) | Null-frameshift | PI 603452 | (Pham et al. 2011) |
| Glyma.20g111000 | *FAD2-1B* | P137R | Missense | PI 283327 | (Pham et al. 2010) |
| Glyma.14g194300 | *FAD3A* | Splice site | Null-splice | CX1512-44 | (Bilyeu et al. 2005) |
|  |  | Deletion | Null-deletion | A5 | (Pham et al. 2014) |
|  |  | W266* | Null-nonsense | C1640 | (Chappell and Bilyeu 2006) |
| Glyma.18g062000 | *FAD3C* | G128E | Missense | CX1512-44 | (Bilyeu et al. 2005) |
|  |  | H304Y | Missense | A29 | (Bilyeu et al. 2006) |

References for Supplemental Table 1:

Bilyeu K, Palavalli L, Sleper D, Beuselinck P (2005) Mutations in soybean microsomal omega-3 fatty acid desaturase genes reduce linolenic acid concentration in soybean seeds. Crop Science 45:1830-1836

Bilyeu K, Palavalli L, Sleper DA, Beuselinck P (2006) Molecular genetic resources for development of 1% linolenic acid soybeans

Crop Science 46:1913-1918

Chappell AS, Bilyeu KD (2006) A *GmFAD3A* mutation in the low linolenic acid soybean mutant C1640. Plant Breed 125:535-536

Pham A-T, Bilyeu K, Chen P, Boerma HR, Li Z (2014) Characterization of the fan1 locus in soybean line A5 and development of molecular assays for high-throughput genotyping of FAD3 genes. Molecular Breeding 33:895-907

Pham A-T, Lee J-D, Shannon J, Bilyeu K (2011) A novel FAD2-1A allele in a soybean plant introduction offers an alternate means to produce soybean seed oil with 85% oleic acid content. TAG Theoretical and Applied Genetics 123:793-802

Pham A-T, Lee J-D, Shannon JG, Bilyeu K (2010) Mutant alleles of FAD2-1A and FAD2-1B combine to produce soybeans with the high oleic acid seed oil trait. BMC Plant Biology 10:195

Online Resource 2

Supplemental Table 2. Details for molecular marker assays for the fatty acid desaturase gene variant alleles utilized

|  |  | Primer | |  |  |  |  |
| --- | --- | --- | --- | --- | --- | --- | --- |
| Gene | Allele | Forward | Reverse | Excess | Simpleprobe (FAM-SPC-sequence-Por) | Cycling | WT/MUT |
| *FAD2-1A* | S117N (-1) | CCAAGGTTGCCTTCTCACTGGT | TAGGCCACCCTATTGTGAGTGTGAC | Forward | GTACTTGCTGAAGGCATGGTGA | 95-20s/65-20s/72-30s | 62/54 |
|  | indel (-2) | CCAAGGTTGCCTTCTCACTGGT | TAGGCCACCCTATTGTGAGTGTGAC | Reverse | CCTCTAGGAAGGGCTGTTTCTCT | 95-20s/65-20s/72-30s | 62/56 |
| *FAD2-1B* | P137R | GGTTCTCCAAGGTTGCATTCTTACT | AGGGTTGTTCAGGTACTTGGTGT | Reverse | AGTCCCTTATTTCTCATGGAAAATAAGC | 95-20s/60-20s/72-20s | 63/57 |
| *FAD3A* | Splice site | TTGCATCACCATGGTCATCAT | AGCTATTATCTAGCATTAACCTCA | Forward | GTTACCTTGCCGCGATACCA | 95-20s/60-20s/72-20s | 64/55 |
|  | W266* | TTGCATCACCATGGTCATCAT | AGCTATTATCTAGCATTAACCTCA | Forward | GTTACCTTGCCGCGATACCA | 95-20s/60-20s/72-20s | 64/59 |
| *FAD3C* | G128E | GTCCTTTGTTGAACAGCATT | CTCCTGCAAAAAATCCATGAGTTGT | Forward | AGGAACCGACCATCCATGGTATGGTACAAGAAT | 95-20s/60-20s/72-20s | 69/64 |
|  | H304Y | TTGGATCAACAACATTCACCA | CATCACATGTTTGTGGTCTTGA | Reverse | TATCCATCACCTTTTCCCTCAAATTC | 95-20s/60-20s/72-20s | 63/56 |

Online Resource 3

Supplemental Table 3. Analysis of Variance results for a total of 21 environments

| Source of variation | Degree of freedom | Palmitic acid | Steric acid | Oleic acid | Linoleic acid | Linolenic acid |
| --- | --- | --- | --- | --- | --- | --- |
| Genotypic group (G) | 5 | 5.4*** | 4.2*** | 242.2*** | 220.3*** | 72.9*** |
| Environment (E) | 20 | 3.9*** | 5.4*** | 67.9*** | 28.5*** | 4.2*** |
| Replication in E | 519 | 0.3*** | 0.2*** | 2.4*** | 1.2*** | 0.1ns |
| G x E | 52 | 1.0*** | 0.3*** | 6.2*** | 4.1*** | 0.4*** |
| Error | 752 | 0.2 | 0.1 | 1.3 | 0.7 | 0.1 |

Online Resource 4

Supplemental Table 4. Fatty acid phenotype mean and standard deviation results for oleic acid and linolenic acid by MG target

| Genotypic groups | MG 00 and 0 | MG I | MG II | MG III | MG IV | MG V | MG VI |
| --- | --- | --- | --- | --- | --- | --- | --- |
|  | Oleic acid (%) | | | | | | |
| HO-1 | . | . | . | 79.3 ± 1.5 | 80.0 ± 1.6 | . | . |
| HO-2 | 83.3 ± 1.2 | 82.3 ± 1.0 | 84.3 ± 1.6 | 83.5 ± 1.2 | 83.2 ± 1.3 | 83.4 ± 1.7 | . |
| HOL-1 | 80.9 ± 2.4 | 81.1 ± 1.6 | . | 80.3 ± 1.3 | 80.8 ± 1.5 | . | 81.4 ± 2.4 |
| HOL-2 | . | 81.9 ± 1.1 | 83.9 ± 1.4 | 83.3 ± 1.2 | 82.9 ± 1.1 | 83.2 ± 1.6 | . |
| HOLL-1 | . | . | 79.3 ± 2.2 | 79.9 ± 2.1 | 80.1 ± 1.9 | . | 81.5 ± 3.2 |
| HOLL-2 | 82.9 ± 0.8 | . | 84.7 ± 1.5 | 83.8 ± 1.1 | 84.0 ± 0.9 | 83.1 ± 1.1 | . |
|  | Linolenic acid (%) | | | | | | |
| HO-1 | . | . | . | 5.0 ± 0.5 | 4.6 ± 0.4 | . | . |
| HO-2 | 4.3 ± 0.5 | 4.5 ± 0.5 | 3.2 ± 0.3 | 3.8 ± 0.5 | 3.6 ± 0.4 | 3.4 ± 0.7 | . |
| HOL-1 | 3.2 ± 0.4 | 3.4 ± 0.3 | . | 3.7 ± 0.6 | 3.6 ± 0.7 | . | 3.0 ± 0.4 |
| HOL-2 | . | 3.6 ± 0.4 | 2.4 ± 0.3 | 3.0 ± 0.3 | 2.9 ± 0.2 | 2.8 ± 0.4 | . |
| HOLL-1 | . | . | 2.0 ± 0.2 | 2.4 ± 0.3 | 2.2 ± 0.2 | . | 2.0 ± 0.3 |
| HOLL-2 | 2.3 ± 0.2 | . | 1.9 ± 0.2 | 2.1 ± 0.2 | 2.0 ± 0.2 | 2.1 ± 0.2 | . |

Online Resource 5

Supplemental Table 5. Environmental stability coefficient (b_E_), and coefficient of determination (*r^2^*) for mean oleic acid and linolenic acid concentrations of six genotypic groups in all environments

| Genotypic group | Oleic acid | | |  | Linolenic acid | | |
| --- | --- | --- | --- | --- | --- | --- | --- |
|  | b_E_ | *P* | *r^2^* |  | b_E_ | *P* | *r^2^* |
| HO-1 | 1.25 | 0.0041 | 0.90 |  | 1.68 | 0.0062 | 0.87 |
| HO-2 | 0.60 | 0.0007 | 0.52 |  | 0.97 | <.0001 | 0.85 |
| HOL-1 | 0.97 | 0.0002 | 0.69 |  | 0.58 | 0.0027 | 0.54 |
| HOL-2 | 0.72 | 0.0009 | 0.61 |  | 0.78 | <.0001 | 0.91 |
| HOLL-1 | 1.08 | 0.0062 | 0.58 |  | 0.41 | 0.0004 | 0.77 |
| HOLL-2 | 0.45 | 0.0365 | 0.29 |  | 0.38 | <.0001 | 0.71 |
